# Supplementary material for: Revisiting Drymaeus germaini (Ancey, 1892) (Gastropoda, Bulimulidae): ecological niche and first anatomical description of a poorly known land snail species from Brazil
Source: PeerJ. 2025 Jul 14;13:e19641. doi: 10.7717/peerj.19641 (PMC12269781; doi:10.7717/peerj.19641)
Supplement: Supplemental Information 1 — The application Gazetteers (https://www.geo-locate.org) was used for the georeferentiation of the localities. When necessary, the coordinates were converted to decimal degree format using Instituto Nacional de Pesquisas espaciais ˗ INPE geographic calculator (http://www.dpi.inpe.br/calcula/). [file peerj-13-19641-s001.docx]

**Supplementary table 1.** Occurrence records of *Drymaeus germaini* (Ancey, 1892) obtained from biodiversity databases.

| **Longitude** | **Latitude** | **Country** | **State** | **County** | **Locality** |
| --- | --- | --- | --- | --- | --- |
| -42,899003 | -20,411368 | Brazil | Minas Gerais | Ponte Nova |  |
| -42,801594 | -20,507743 | Brazil | Minas Gerais | Ponte Nova | Amparo da Serra |
| -42,863022 | -20,759782 | Brazil | Minas Gerais | Viçosa | Recanto das Cigarras UFV |
| -46,641681 | -23,573252 | Brazil | São Paulo | São Paulo |  |
| -50,00206 | -24,798294 | Brazil | Paraná | Casiro |  |
| -52,338858 | -22,855059 | Brazil | Paraná | São João do Caiuá | Fazenda Santa Rita de Cassia |
| -42,871445 | -20,760193 | Brazil | Minas Gerais | Viçosa | Campus UFV |
| -42,392247 | -19,352650 | Brazil | Minas Gerais | Ipaba | Fazenda Macedônia |
| -55,926497 | -9,596857 | Brazil | Mato Grosso | Novo Mundo |  |
| -55,931865 | -9,597604 | Brazil | Mato Grosso | Novo Mundo |  |
| -55,921565 | -9,555626 | Brazil | Mato Grosso | Alta Floresta |  |
| -47,596354 | -21,723096 | Brazil | São Paulo | Santa Rita do Passa-Quatro |  |
| -42,863013 | -20,757842 | Brazil | Minas Gerais | Recanto das Cigarras UFV |  |
| -42,392768 | -19,360155 | Brazil | Minas Gerais | Ipaba |  |
| -54,913521 | -22,210872 | Brazil | Mato Grosso do Sul | Dourados | Unamed Road |
| -55,51026 | -11,854378 | Brazil | Mato Grosso | Sinop |  |
| -55,935444 | -9,585539 | Brazil | Mato Grosso | Alta Floresta |  |
| -65.333333 | -10.816667 | Brazil | Rondônia | Guayará-Mirim | 284 km above Porto Velho, Mamoré Rail Road |
| -64.423003 | -12.425667 | Brazil | Rondônia | Forte Príncipe da Beira |  |
| -59,94852 | -15,00692 | Brazil | Mato Grosso | Maracaju |  |
